# Validated Prognostic Scores to Predict Outcomes in ECLS-Bridged Patients to Lung Transplantation

**DOI:** 10.3389/ti.2023.11609

**Published:** 2023-10-30

**Authors:** Eleonora Faccioli, Giulia Lorenzoni, Didier Schneiter, Andrea Dell’Amore, Sven Hillinger, Marco Schiavon, Claudio Caviezel, Dario Gregori, Federico Rea, Isabelle Opitz, Ilhan Inci

**Affiliations:** ^1^ Thoracic Surgery Unit, Department of Cardiac, Thoracic Vascular Sciences and Public Health, University Hospital of Padua, Padua, Italy; ^2^ Department of Thoracic Surgery, University Hospital of Zurich, Zurich, Switzerland; ^3^ Unit of Biostatistics, Epidemiology and Public Health, Department of Cardiac, Thoracic Vascular Sciences and Public Health University of Padua, Padua, Italy

**Keywords:** outcomes, lung transplantation, bridge to transplant, SOFA, extracorporeal life support

## Abstract

Selection of patients who may benefit from extracorporeal life support (ECLS) as a bridge to lung transplant (LTx) is crucial. The aim was to assess if validated prognostic scores could help in selecting patients who may benefit from ECLS-bridging predicting their outcomes. Clinical data of patients successfully ECLS-bridged to LTx from 2009 to 2021 were collected from two European centers. For each patient, we calculated Sequential Organ Failure Assessment (SOFA), Simplified Acute Physiology Score III (SAPS III), Acute Physiology and Chronic Health Evaluation II (APACHE II), before placing ECLS support, and then correlated with outcome. Median values of SOFA, SAPS III, and APACHE II were 5 (IQR 3–9), 57 (IQR 47.5–65), and 21 (IQR 15–26). In-hospital, 30 and 90 days mortality were 21%, 14%, and 22%. SOFA, SAPS III, and APACHE II were analyzed as predictors of in-hospital, 30 and 90 days mortality (SOFA C-Index: 0.67, 0.78, 0.72; SAPS III C-index: 0.48, 0.45, 0.51; APACHE II C-Index: 0.49, 0.45, 0.52). For SOFA, the score with the best performance, a value ≥9 was identified to be the optimal cut-off for the prediction of the outcomes of interest. SOFA may be considered an adequate predictor in these patients, helping clinical decision-making. More specific and simplified scores for this population are necessary.

## Graphical Abstract

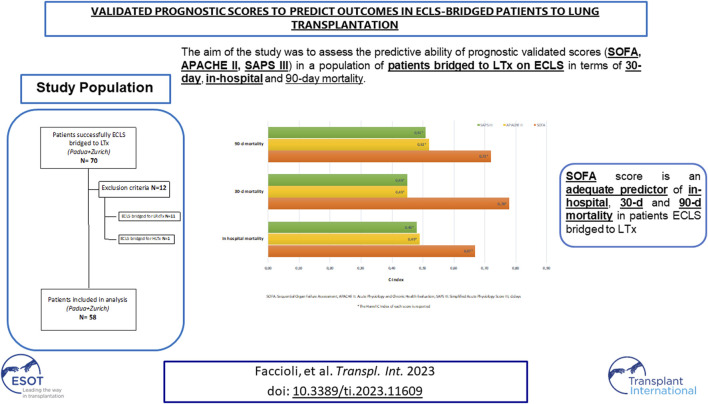

## Introduction

The utilization of extracorporeal life support (ECLS) as a bridge to lung transplantation (LTx) has allowed critically ill patients to remain eligible for transplant.

The selection of patients who may benefit from ECLS as a bridge to LTx is a crucial aspect: highly urgent patients, with a high predicted pre-transplant mortality, are often the ones who would benefit the most from ECLS but at the same time they could be too compromised to be suitable candidates for this support [1].

The patients who can derive the greatest benefit from ECLS-bridge are generally those with cardiopulmonary dysfunction severe enough to limit their ability to maintain the necessary physical condition to tolerate a transplant (such as oxygen saturation <90% with high-flow levels and with non-invasive oxygenation devices, hemodynamic instability, and use of positive pressure ventilation that could lead to further lung injury and secondary organ dysfunctions) and it is mostly recommended in patients who have already been evaluated for LTx [1–4].

The effect of ECLS as a bridge to LTx and the consequences on recipients’ clinical outcomes remain undetermined, indeed the results reported in current literature are divergent [5, 6].

Some authors [7, 8] reported negative experiences with ECLS as a bridge to LTx, showing a worse overall survival in bridged patients compared to unsupported ones. On the other hand, in more recent times, different authors have reported good outcomes for successfully bridged patients on ECLS with satisfying survival rates [2, 3, 9–11].

It is widely established that a careful patient selection, high volume transplant centers, and multidisciplinary teams are the key factors to obtain improvements in ECLS bridging strategies [1], even though a homogeneous consensus on which factors might help the clinicians in predicting outcomes of patients bridged to LTx with ECLS supports is still lacking. This is also demonstrated by the fact that currently no clinically validated tools, except the Recipient STratification Risk Analysis in Bridging Patients to Lung Transplant on ECMO (STABLE) score [12], exist to predict outcomes in this population. However, given the increasing use of different bridging devices (not only extracorporeal membrane oxygenation, ECMO) and strategies in the modern era, it is mandatory to define if validated prognostic scores might predict mortality in this population, helping to better select patients who may benefit from ECLS bridging in relation to post-operative outcomes.

Scoring systems, such as Sequential Organ Failure Assessment (SOFA), Simplified Acute Physiology Score III (SAPS III), and Acute Physiology and Chronic Health Evaluation II (APACHE II), are commonly used for risk assessment in critically ill patients, especially to predict in-hospital mortality [13–15].

The aim of this study was to assess the predictive ability of these scores in a population of patients bridged to LTx on ECLS in terms of in-hospital, 30 and 90 days mortality.

These findings might play an important role in guiding physician decision making to better select patients who might benefit from a bridge from ECLS to LTx, also facilitating evidence-based rationing of limited healthcare resources in the future.

## Materials and Methods

All clinical data of 70 patients successfully ECLS-bridged to LTx from 2009 to 2021 were retrospectively collected from two European centers (Thoracic Surgery Unit of University Hospital of Padua, Italy and Department of Thoracic Surgery of University Hospital of Zurich, Switzerland) as anonymized records. The study was approved by the Ethical Committee of the University Hospital of Padua (4539/AO/18). Informed consent was waived due to the retrospective nature of this work.

**FIGURE 1:**
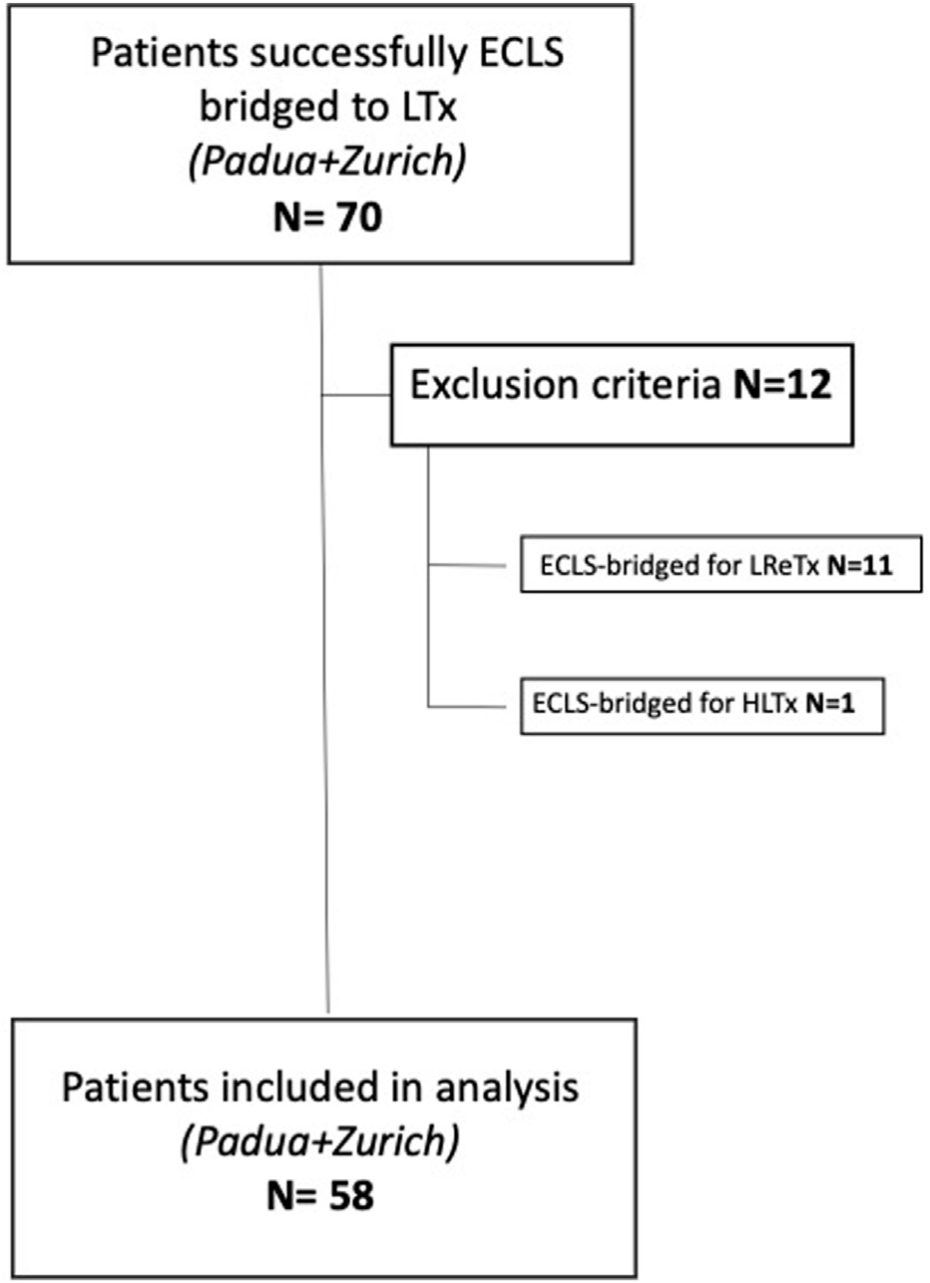
Flowchart of patients’ inclusion and exclusion criteria (ECLS, extracorporeal life support; LTx, lung transplant; LreTx, lung re-transplant; HLTx, heart-lung transplant).

Patients bridged to lung retransplantation (LReTx) or heart-lung transplantation (HLTx) with ECLS were excluded. Fifty-eight patients were finally enrolled in the study (Figure 1).

Demographic and clinical data, intra-operative characteristics, peri and post-operative outcomes were collected for each patient from both centers. Follow-up was achieved in each center by indirect contact via the treating physician. For each patient, organ dysfunction (SOFA) and illness severity (APACHE II, SAPS III) scores were collected when already available otherwise calculated retrospectively at the ICU arrival, before positioning the ECLS device and then correlated with outcomes. A comparison between the variables utilized in the abovementioned scoring systems is shown in Table 1.

**TABLE 1:** Variables employed in SOFA, APACHE II, SAPS III scoring systems.

	SOFA (range 0–20)	APACHE II (range 0–71)	SAPS III (range 16–217)
Variables	- PaO2/FiO2 (and if MV/CPAP)	- Temperature	- Age
	- Platelets	- Age	- LOS before ICUA
	- GCS	- MAP	- In- hospital location (OR, ER, other ICU)
	- Bilirubin	- HR	- Cancer therapy (yes/no)
	- MAP (and if vasoactive agents required)	- RR	- Chronic heart failure (yes/no)
	- Creatinine	- pH	- Hematological cancer (yes/no)
		- Sodium	- Cirrhosis (yes/no)
		- Potassium	- AIDS (yes/no)
		- Creatinine	- Cancer (yes/no)
		- Hematocrit	- Vasoactive drugs before ICUA (yes/no)
		- WBC	- ICUA (planned/unplanned)
		- Chronic organ failure (heart, lung, liver, kidney)	- Reason for admission (cardiovascular, hepatic, digestive, neurologic)
		- GCS	- Surgical status at ICUA (scheduled, emergency, no surgery)
		- FiO2	- site of surgery (transplant, trauma, cardiac, neurosurgery)
			- acute infection at ICUA (nosocomial, respiratory)
			- GCS
			- Bilirubine
			- Temperature
			- Creatinine
			- HR
			- WBC
			- pH
			- Platelets
			- SBP
			- pO2/FiO2
			- MV (yes/no/CPAP)

APACHE II, acute physiology and chronic health evaluation II; CPAP, continuous positive pressure ventilation; ER, emergency room; FiO2, fraction of inspired oxygen; GCS, Glasgow coma scale; HR, heart rate; ICU, intensive care unit; ICUA, intensive care unit admission; LOS, length of stay; MAP, mean arterial pressure; MV, mechanical ventilation; OR, operative room; paO2, partial pressure of oxygen; RR, respiratory rate; WBC, white blood cells; SBP, systolic blood pressure; SAPS III, simplified acute physiology score III; SOFA, sequential organ failure assessment.

### Statistical Analyses

Descriptive statistics were reported as I quartile/median/III quartile for continuous variables and as percentages (absolute numbers) for categorical variables.

Survival distribution was evaluated using the Kaplan-Meier method. To assess the predictive ability of the scores (SOFA, APACHE II, SAPS III) on the outcomes of interest (in-hospital, 30 and 90 days mortality) logistic regression models were estimated. After models’ validation using bootstrap resampling, the Harrel’s C index, also known as “concordance index” [16] was computed.

Furthermore, the optimal cut-off for SOFA in predicting outcomes of interest was identified as the value that maximizes the sum of sensitivity and specificity.

Analyses were performed using R software version 4.1.3 [17] within the packages rms [18] and cutpointr [19].

## Results

### Study Population

The main clinical and demographic characteristics of 58 patients bridged to LTx on ECLS support are presented in Table 2 *.*

**TABLE 2:** Patients characteristics.

Variable	Total (N = 58)
Female sex	34 (59%)
Age at LTx (y)	42 (24–49)
Diagnosis	
CF	33 (57%)
ILD	16 (27%)
COPD	4 (7%)
LAM	1 (2%)
Other	4 (7%)
BMI	19.5 (17–24)
Waiting list time (d)	69 (14–240)
Type of LTx	
BLTX	57 (98%)
SLTX	1 (2%)
Pre LTx MV	
No	10 (17%)
Yes	48 (83%)
Awake ECLS	10 (17%)
Time from ECLS bridge to LTx (d)	10 (3–18)
Initial ECLS bridge configuration	
VV-ECMO	37 (64%)
VA-ECMO	8 (14%)
ECCO2-R	13 (22%)
ECLS bridge configuration change	18 (30%)
SOFA	5 (3–9)
pSOFA (age <18 y)	5 (3–10)
APACHE II	21 (15–26)
SAPS III	57 (47.5–65)
Intraoperative ECLS configuration	
VV-ECMO	18 (31%)
pVA-ECMO	12 (21%)
cVA-ECMO	15 (26%)
CEC	4 (7%)
VAV	9 (15%)
Prolonged post-operative ECLS	
NO	28 (47%)
YES	30 (51%)
*De novo*	1 (2%)
Post-operative ECLS duration (d)	3 (2–8)
EVLP	4 (7%)
Lobar transplantation	17 (29%)

APACHE II, Acute Physiology and Chronic Health Evaluation II; BLTX, bilateral lung transplant; c, central; d, days; ECCO2-R, extracorporeal carbon dioxide removal; ECLS, extracorporeal life support; ECMO, extracorporeal membrane oxygenation; EVLP, *ex-vivo* lung perfusion; f, female; LTx, lung transplant; MV, mechanical ventilation; p, peripheral; SOFA, Sequential Organ Failure Assessment; SAPS III, Simplified Acute Physiology Score III; y, years; VA, venoarterial; VAV, venoarterial venous; VV, venovenous.

Data are reported as median (I-III interquartile range) for continuous variables and as absolute number (relative frequencies %) for categorical variables.

Thirty-four patients (59%) were females, 24 were males (41%) with a median age at time of LTx of 42 years-old (IQR 24–49). Seven patients (12%) were in pediatric age (age <18 years). The median BMI was 19.5 (IQR 17–24). The most common indication for LTx was cystic fibrosis (CF) (57%) followed by interstitial lung disease (ILD) (27%); almost all patients (98%) underwent bilateral lung transplantation while only a 65 year-old patient affected by ILD was submitted to single LTx.

The median waiting list time was 69 days (IQR 14–240). The most common ECLS bridge configuration was veno-venous (VV) (37 patients, 64%) although in 30% of cases an upgrading to another ECLS configuration was necessary during bridging. During ECLS bridging, 48 patients (83%) were mechanically ventilated while 10 patients (17%) were awake. The median time from ECLS bridge to LTx was 10 days (IQR 3–18). Median SOFA, APACHE II, and SAPS III values at the ICU arrival were respectively 5 (IQR 3–10), 21 (IQR 15–26), 57 (IQR 47.5–65). Median pediatric (p) SOFA, a special score tailored on pediatric patients [19], was also calculated but the median value did not differ from the one obtained in adults (5, IQR 3–10). The most common intra-operative ECLS configuration was the VV (18 patients, 31%) followed by the central veno-arterial (VA) ECLS (15 patients, 26%). 30 patients (51%) needed prolonged post-operative ECLS with a median duration of 3 days (IQR 2–8).

In 4 cases (7%) *ex-vivo* lung perfusion (EVLP) methods were used to recondition the organs before the implantation because of extended criteria donors and in 17 patients (29%) a size reduction with lobar LTx was performed due to the donor and the recipient size mismatch.

### Short-Term Outcomes

Table 3 summarizes the main post-operative outcomes. The median duration of mechanical ventilation (MV) was 96 h (IQR 48–480) and in 28 patients (48%) a tracheostomy was performed for respiratory weaning. The median duration of ICU and hospital stay were respectively 11 days (IQR 6–28) and 44 days (IQR 31–71). Twenty-four patients (41%) required post-operative continuous veno-venous hemofiltration (CVVH) or dialysis for renal failure. In-hospital, 30, and 90 days mortality were respectively 21%, 14%, and 22%.

**TABLE 3:** Clinical course and outcomes.

Variable	Total (N = 58)
ICU stay (post LTx, d)	11 (6–28)
MV duration (post LTx, h)	96 (48–480)
Post-operative tracheostomy	28 (48%)
Post-operative CVVH/dialysis	24 (41%)
Hospital stay (d)	44 (31–71)
CLAD	8 (14%)
In hospital mortality	12 (21%)
30 d mortality	8 (14%)
90 d mortality	13 (22%)

CLAD, chronic lung allograft dysfunction; CVVH, continous venovenous hemofiltration; d, days; h, hours; ICU, intensive care unit; LTx, lung transplant; MV, mechanical ventilation.

Data are reported as median (I-III interquartile range) for continuous variables and as absolute number (relative frequencies %) for categorical variables.

### Long-Term Outcomes

**FIGURE 2:**
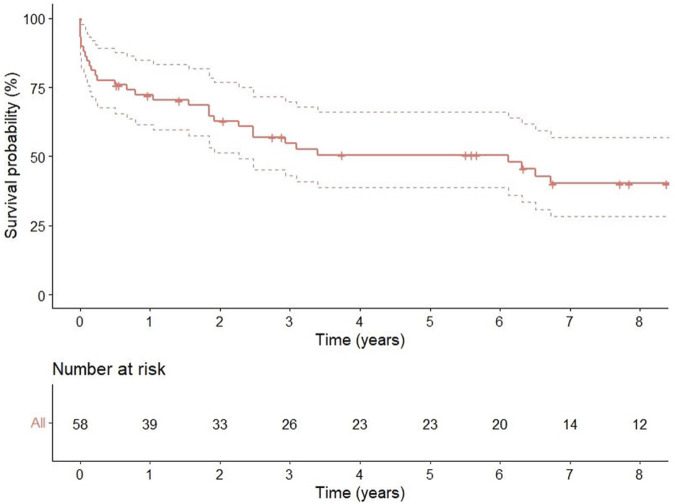
Kaplan-Meyer curve of the overall survival in ECLS-bridged patients.

One, 3, and 5 years survival rates were 72 % (95% CI 0.61–0.84), 55% (95% CI 0.43–0.70), and 51% (95% CI 0.38–0.66), respectively (Figure 2).

### Predictive Ability of SOFA, APACHE II, and SAPS III

The ability of SOFA, APACHE II, and SAPS III in predicting post-LTx outcomes in ECLS-bridged patients is presented as C Index in Table 4 *.*

**TABLE 4:** Predictive ability of SOFA, APACHE II, SAPS III scores in predicting outcomes.

Score	Outcomes			
	In hospital mortality	30 d mortality	60 d mortality	90 d mortality
SOFA	0.67	0.78	0.60	0.72
APACHE II	0.49	0.45	0.42	0.52
SAPS III	0.48	0.45	0.42	0.51

APACHE II, Acute Physiology and Chronic Health Evaluation II; d, days; SAPS III, Simplified Acute Physiology Score III; SOFA, Sequential Organ Failure Assessment.

The Harrel’s C Index of each score for in-hospital, 30, 60, and 90 days mortality is reported.

SOFA, SAPS III, and APACHE II were analyzed as predictors of in-hospital, 30, and 90 days mortality respectively (SOFA C Index: 0.67, 0.78, 0.72; SAPS III C Index: 0.48, 0.45, 0.51; APACHE II C Index: 0.49, 0.45, 0.52). For SOFA, the score with the best performance in this population, a value ≥9, was identified to be the optimal cut-off for the prediction of all the outcomes of interest (Table 5).

**TABLE 5:** Performance of SOFA value ≥9.

Outcome	Sensitivity	Specificity	AUC
In-hospital mortality	0.714	0.780	0.775
30 d mortality	0.6	0.789	0.680
90 d mortality	0.636	0.810	0.717

AUC, area under curve; d, days.

## Discussion

Patients bridged to LTx with ECLS are often critically ill with a severe deterioration of clinical conditions. The investigation of predictors of outcomes in this population is mandatory, especially in a context of donors’ paucity as well as to facilitate evidence-based rationing of limited healthcare resources in the future.

We decided to analyze the predictive ability of three scores (SOFA, APACHE II, SAPS III), which are widespread known and easily accessible for every patient at the ICU arrival, to predict post-operative outcomes in ECLS bridged patients. These scores have already been extensively validated as predictors of mortality in several clinical settings [20–22] including transplantation field [23] and in patients on ECMO for cardiac or acute respiratory failure [24, 25]. However, they were not validated in ECLS-bridged patients to LTx. In addition, a number of specific scores in VA ECMO settings like prEdictioN of Cardiogenic shock OUtcome foR AMI patients salvaGed by VA ECMO (ENCOURAGE), Survival After Veno-arterial ECMO (SAVE), and pRedicting mortality in patients undergoing veno-arterial Extracorporeal MEMBrane oxygenation after coronary artEry bypass gRafting (REMEMBER) have been proposed to predict mortality in selective cardiogenic shock subsets [26–28], limiting their application in our study population.

On the other hand, The Respiratory Extracorporeal Membrane Oxygenation Survival Prediction (RESP) Score predicts survival for patients receiving ECMO for severe acute respiratory failure [29] but again it is not tailored to chronic end-stage lung disease and it does not take into account those patients with an associated hemodynamic instability (like in idiopathic pulmonary hypertension).

To the best of our knowledge, the only available predictive tool for risk stratification in ECMO bridge patients to LTx is the STABLE score [12] but some of its limitations made it not applicable to our entire population: firstly, it is validated only in adults but our population was composed for 12% of pediatric patients. In our study, in accordance with what has been reported by some of the most consistent studies on ECLS bridge [2, 8], more than a half (57%) of our population had cystic fibrosis which is the most common indication in pediatric population, therefore a score also applicable in a pediatric population (<18 years old) is mandatory. In our pediatric patients, we have also calculated for each of them the pSOFA, an adapted and validated pediatric version of the SOFA score [20], finding the same median value of the adult population. Concerning the other two scores, APACHE II has already been utilized for pediatrics in other clinical setting [30] and in the SAPS III calculator, ages of <18 years old can be inserted, so we felt authorized to use these scores also in pediatrics.

Secondly, among extracorporeal supports, the STABLE score only considers ECMO and not other devices such as extracorporeal carbon dioxide removal (ECCO2-R), which was utilized in 22% of our population as a bridge to LTx. Finally, this score was created on a big number of patients extracted from United Network for Organ Sharing (UNOS) database but it was externally validated only on 31 American patients and so it could not be representative of the European reality.

Among the three scores utilized in our analysis, the predictor of in-hospital, 30- and 90-days mortality with the best performance was the SOFA with a cut-off value of 9. SOFA score is the easiest to calculate and based on easy repeatable variables available in all institutions. Originally, it was designed to describe morbidity expressing different degrees of organ failure, but then it has been extensively externally validated as a good predictor of hospital mortality [14, 25].

In contrast to our finding [24], in a previous study, compared the prognostic ability of different scores in ECMO patients, showing that APACHE had a superior ability to SOFA in predicting hospital mortality. Their study did not focus on patients bridged to LTx and furthermore the scores were calculated only on the first day of ECMO support and not at the ICU admission as these models were originally developed and this may have affected the results. The low accuracy of APACHE II and SAPS III in predicting in-hospital mortality in transplant patients has already been established [23]. We also reported the same finding in ECLS bridged patients to LTx; this may be due to the multitude of physiologic aspects (such as for examples sodium, potassium, hematocrit, white blood cells, and platelets count) accounted by these two scores compared to the SOFA. These parameters are usually out of normality range in this population and tend to have a large and rapid variability during the pre- and post-transplant periods, making these scores unreliable in our patients.

Although in our study, a SOFA score of higher than or equal to 9 was associated with a poor short-term prognosis, this value should not be intended to arbitrarily exclude patients from life-sustaining therapies or from the possibility of a lung transplantation but just as a useful tool to better select the most appropriate LTx candidate or to help clinicians to identify which patients would need a stricter follow-up in the early post-operative period.

In conclusion, the results of this study serve as a first external validation of these scores in ECLS-bridged patients to LTX but it has some limitations. Even though it reflects the reality of two European lung transplant centers, the main limitation is the small number of patients and the absence of a control group. Then, in some situations, difficulties exist in performing this analysis as clinical/laboratory data to calculate the scores are not always collected at the same moment for all the subjects with a tendency towards high variability during pre-operative course during ECLS bridging. Again, it would be necessary to evaluate the evolution of these scores in different moments by sequential measurements (daily, weekly) and not only at the ICU admission, providing a more robust prediction of mortality.

Further studies are necessary to validate our results and to find a promising and accurate score in this peculiar subgroup of patients.

## Data Availability

The raw data supporting the conclusion of this article will be made available by the authors, without undue reservation.

## Abbreviations

APACHE II, Acute Physiology and Chronic Health Evaluation II; CIFs, cumulative incidence functions; CLAD, chronic lung allograft dysfunction; ECCO2-R, extracorporeal carbon dioxide removal; ECLS, extracorporeal life support; ECMO, extracorporeal membrane oxygenation; ENCOURAGE, prEdictioN of Cardiogenic shock OUtcome foR AMI patients salvaGed by VA ECMO; EVLP, *ex-vivo* lung perfusion; HLTx, heart-lung transplantation; ICU, intensive care unit; IQR, interquartile range; MV, mechanical ventilation; LTx, lung transplantation; LreTx, lung retransplantation; pSOFA, pediatric Sequential Organ Failure Assessment; REMEMBER, pRedicting mortality in patients undergoing veno-arterial Extracorporeal MEMBrane oxygenation after coronary artEry bypass gRafting; RESP, Respiratory Extracorporeal Membrane Oxygenation Survival Prediction; SAPS III, Simplified Acute Physiology Score III; SAVE, Survival After Veno-arterial ECMO; SOFA, Sequential Organ Failure Assessment; STABLE, Recipient STratification Risk Analysis in Bridging Patients to Lung Transplant on ECMO; UNOS, United Network for Organ Sharing; VV, veno-venous; VA, veno-arterial.
